# Statistical modeling for bioconvective tangent hyperbolic nanofluid towards stretching surface with zero mass flux condition

**DOI:** 10.1038/s41598-021-93329-y

**Published:** 2021-07-06

**Authors:** Anum Shafiq, S. A. Lone, Tabassum Naz Sindhu, Q. M. Al-Mdallal, G. Rasool

**Affiliations:** 1 School of Mathematics and Statistics, Nanjing University of Information Science and Technology, Nanjing, 210044 China; 2 Department of Basic Science, College of Science and Theoretical Studies, Saudi Electronic University, Riyadh, Kingdom of Saudi Arabia; 3 Department of Statistics, Quaid-i-Azam University 4250, Islamabad, 44000 Pakistan; 4 Department of Mathematical Sciences, UAE University, P.O. Box 15551 Al-Ain, United Arab Emirates; 5 Binjiang College, Nanjing University of Information Science and Technology, Wuxi, 214105 Jiangsu China

**Keywords:** Mathematics and computing, Applied mathematics

## Abstract

This article presents the implementation of a numerical solution of bioconvective nanofluid flow. The boundary layer flow (BLF) towards a vertical exponentially stretching plate with combination of heat and mass transfer rate in tangent hyperbolic nanofluid containing microorganisms. We have introduced zero mass flux condition to achieve physically realistic outcomes. Analysis is conducted with magnetic field phenomenon. By using similarity variables, the partial differential equation which governs the said model was converted into a nonlinear ordinary differential equation, and numerical results are achieved by applying the shooting technique. The paper describes and addresses all numerical outcomes, such as for the Skin friction coefficients (SFC), local density of motile microorganisams (LDMM) and the local number Nusselt (LNN). Furthermore, the effects of the buoyancy force number, bioconvection Lewis parameter, bioconvection Rayleigh number, bioconvection Pecelt parameter, thermophoresis and Brownian motion are discussed. The outcomes of the study ensure that the stretched surface has a unique solution: as *Nr* (*Lb*) and *Rb* (*Pe*) increase, the drag force (mass transfer rate) increases respectively. Furthermore, for least values of *Nb* and all the values of Nt under consideration the rate of heat transfer upsurges. The data of SFC, LNN, and LDMM have been tested utilizing various statistical models, and it is noted that data sets for SFC and LDMM fit the Weibull model for different values of *Nr* and *Lb* respectively. On the other hand, Frechet distribution fits well for LNN data set for various values of *Nt*.

## Introduction

Recently, several studies have been conducted on stretching surfaces, that are used in industrial materials like glass fibers and lubricants. Crane^1^ suggested the flowing mechanisms towards a stretched surface. Investigators^2^ have studied heat transfer phenomenon using permeable stretching sheet. Numerous other researchers performed similar studies involving a stretching surface (see^3–8^). Convective heat transfer is a significant feature of nanofluids, and it has been found that incorporating nanomaterials enhances the thermal conductivity. Nanofluids have received extensive interest of recent investigators because of their numerous potential usages like in power generation, nuclear reactors, electronics, biomedicine, chemical processes, space technology and nanotechnology. In^9^, Makinde and Aziz analyzed boundary layer (BL) stream of nanoliquid towards a stretched plate via CBC (convective boundary conditions). In^10^, combined impacts of heat, mass phenomena in stream of nanoliquids towards a non-horizontal surface via radiation is scrutinized. In^11^, Mustafa et al. investigated unsteady BL flow of nanoliquid towards a stretching surface. In^12^, Ashorynejad et al. analyzed heat transfer characteristics of nanoliquid by incorporating MHD effect. Murthy et al.^13^ examined convection heat transfer phenomenon in stratified nanoliquid under non-Darcy porous phenomenon. The formulation of entropy generation using nanoliquid via rotating porous plate was reported by Rashidi et al.^14^. Jedi et al.^15^ studied statistical modeling of nanofluid flow towards the stretching surface. They gave the concept of modeling the data of considered studied statistically via incorporating statistical distributions. Chu et al.^16,17^ studied ANN modeling of nanofluid examined experimentally and then the results were compared with regression-based methodologies.

Bioconvection has various uses in biological and biotechnological processes. The bioconvection term indicates a macroscopic convective movement of liquid induced by density gradient produced due to joint swimming of motile microorganisms. By moving in a specific direction, such self-propelled motile microorganisms rise density of base liquid, thereby initiating bioconvection. The bioconvection process in nanofluid convection is associated with presence of denser microorganisms that accumulate on lighter water surface. As heavier microbes fall into water, up-swimming microbes replenish them, thus creating the mechanism of bioconvection within system. The mechanism is a mesoscale phenomenon where a macroscopic movement is caused by motion of motile micro-organisms (MMs). Nanomaterials are not self-propelled unlike motile microorganisms. Their movement is driven by thermophoresis and Brownian phenomena happening in nanofluid. Therefore, movement of MMs (motile micro-organisms) is free of movement of nanoparticles. The addition of micro-organisms to a nanofluid improves its stability as a suspension^18^ and may prevent aggregation and agglomeration of nanoparticles. In^19^, Aziz et al. studied free convective BL flow over a horizontal surface in nanoliquid including gyrotactic microorganisms. They noted that bioconvective numbers significantly influenced mass, motile micro-organism and heat transfer rate. In^20^, Tham et al. numerically examined mixed convective BL flow about a solid surface saturated in porous medium via nanoliquid including gyrotactic microorganisms by considering heated and cooling sphere. In^21^ Ibrahim studied the time-dependent viscous fluid flow due to a rotating stretchable disk.

A comprehensive explanation^22–34^ is given for onset of bioconvection in suspension of oxytactic/gyrotactic micro-organisms in different situations.

Motivated by Jedi et al.^15^, we have investigated the BLF of tangent hyberbolic nanoliquid containing gyrotactic microorganisms with zero mass flux condition. Our main aim here is to find effect of key numbers (buoyancy force parameter, bioconvection Rayleigh parameter, thermophoresis, Brownian motion, bioconvective Lewis number and bioconvective Pecelt number). The shooting methodology along with RK4 has utilized to gain the outcomes for SFC, LNN and LDMM. In order to estimate thermal conductivity of a nanoliquid containing microorganisms, a physical-statistical model, as well as its distribution is considered. In further research on nanoliquids containing microorganisms, the proposed model could be used for a wide variety of practical uses.

## Formulation

**Figure 1:**
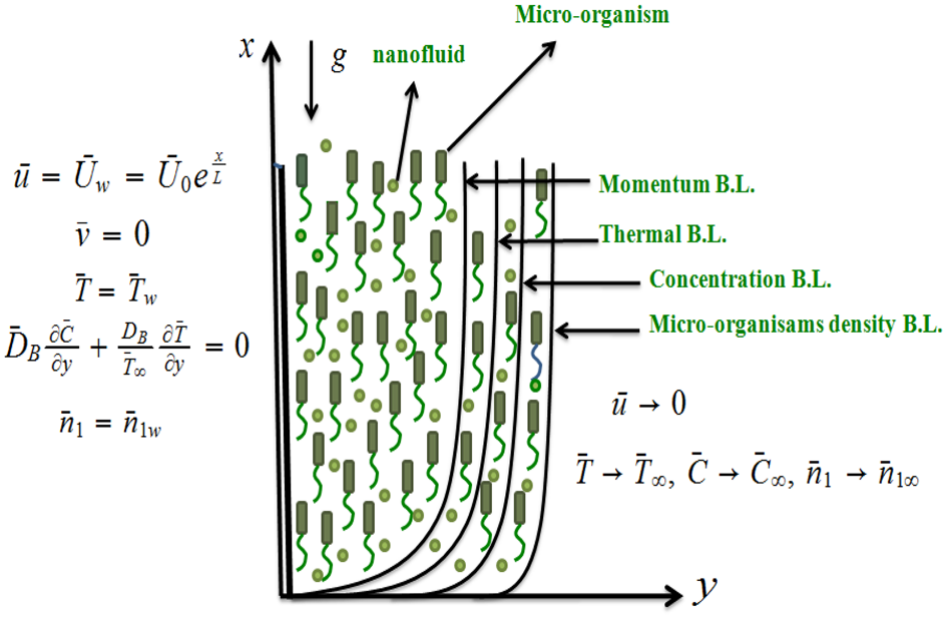
Physical model.

The steady BL flow of tangent hyperbolic nanoliquid containing microorganisams over a vertically exponential stretching plate with zero mass flux condition is considered. The MHD and Joule heating phenomena in the absence of viscous dissipation is considered into account. The physical configuration scheme is illustrated in Fig. 1. The current flow is driven by following set of equations^26,27^:

1 $$\begin{aligned}&\frac{\partial {\bar{u}}}{\partial x}+\frac{\partial {\bar{v}}}{\partial y}=0, \end{aligned}$$

2 $$\begin{aligned}&{\bar{u}}\frac{\partial {\bar{u}}}{\partial x}+{\bar{v}}\frac{\partial {\bar{u}}}{ \partial y} =\upsilon (1-n)\frac{\partial ^{2}{\bar{u}}}{\partial y^{2}} +2\upsilon \Gamma n\frac{\partial {\bar{u}}}{\partial y}\frac{\partial ^{2} {\bar{u}}}{\partial y^{2}}-\frac{\sigma }{{\bar{\rho }}}B_{0}^{2}{\bar{u}}+\frac{1}{ {\bar{\rho }}}\left[ (1-{\bar{C}}_{\infty }){\bar{\rho }}\beta _{T}g({\bar{T}}-{\bar{T}} _{\infty })\right. \nonumber \\&\left. -\left( {\bar{\rho }}_{p}-{\bar{\rho }}\right) g(C-C_{\infty })-\left( {\bar{n}}_{1}-{\bar{n}}_{1\infty }\right) g\gamma \left( {\bar{\rho }}_{m}-{\bar{\rho }}\right) \right] , \end{aligned}$$

3 $$\begin{aligned}&u\frac{\partial {\bar{T}}}{\partial x}+v\frac{\partial {\bar{T}}}{\partial y}= \frac{\kappa }{\rho c_{p}}\frac{\partial ^{2}{\bar{T}}}{\partial y^{2}}+{\bar{\tau }}\left[ D_{B}\frac{\partial {\bar{T}}}{\partial y}\frac{\partial C}{ \partial y}+\frac{D_{T}}{T_{\infty }}\left( \frac{\partial {\bar{T}}}{\partial y}\right) ^{2}\right] +\frac{\sigma }{\rho c_{p}}{\bar{B}}_{0}^{2}{\bar{u}}^{2}, \end{aligned}$$

4 $$\begin{aligned}&{\bar{u}}\frac{\partial C}{\partial x}+{\bar{v}}\frac{\partial C}{\partial y}= \frac{D_{T}}{T_{\infty }}\frac{\partial ^{2}{\bar{T}}}{\partial y^{2}}+D_{B} \frac{\partial ^{2}C}{\partial y^{2}}, \end{aligned}$$

5 $$\begin{aligned}&{\bar{u}}\frac{\partial n_{1}}{\partial x}+{\bar{v}}\frac{\partial n_{1}}{ \partial y}+\frac{{\bar{b}}W_{c}}{\left( C_{w}-C_{\infty }\right) }\frac{ \partial }{\partial y}\left( n_{1}\frac{\partial C}{\partial y}\right) =D_{m} \frac{\partial ^{2}n_{1}}{\partial y^{2}}, \end{aligned}$$

with

6 $$\begin{aligned}&{\bar{u}}={\bar{U}}_{w}={\bar{U}}_{0}e^{\frac{x}{L}},\text { }{\bar{v}}=0,\text { } {\bar{T}}={\bar{T}}_{w},\text { }D_{B}\frac{\partial C}{\partial y}+\frac{D_{B}}{ {\bar{T}}_{\infty }}\frac{\partial {\bar{T}}}{\partial y}=0,\text { }n_{1}=n_{1w} \text { at }y=0, \nonumber \\&{\bar{u}}\rightarrow 0,\text { }{\bar{T}}\rightarrow {\bar{T}}_{\infty },\text { } C\rightarrow C_{\infty },\text { }n_{1}\rightarrow n_{1\infty }\text { when } y\rightarrow \infty . \end{aligned}$$

Here velocity components $\left( {\bar{u}},{\bar{v}}\right)$ in $\left( x,y\right)$ directions respectively, ${\bar{\rho }}$ density of nanoliquid, $\mu$ viscosity of nanoliquid and microorganisms, density of nanomaterials is ${\bar{\rho }}_{p}$, electrical conductivity of nanoliquid is $\sigma$, density of microorganisms materials ${\bar{\rho }}_{m}$, heat capacity ratio of nanomaterials by nanoliquid is ${\bar{\tau }}=\frac{\left( {\bar{\rho }}c\right) _{p}}{\left( {\bar{\rho }}c\right) _{f}}$, temperature of liquid is ${\bar{T}}$, density motile of microorganisms is $n_{1}$, concentration of nanomaterials *C*, kinematic viscosity $\upsilon$, volume expansion coefficient of liquid $\beta _{T}$, gravity is *g*, average volume of a micro-organism $\gamma$, specific heat $c_{p}$, ${\bar{U}}_{w}$ is the stretching velocity, chemotaxis constant ${\bar{b}}$ and maximum cell swimming speed $W_{c}$, thermophoretic diffusion coefficient $D_{T}$, Brownian motion diffusion coefficient $D_{B}$ , ambient temperature ${\bar{T}}_{\infty }$, ambient concentration of nanoparticles $C_{\infty }$, ambient microorganisms concentration $n_{1\infty }$. and $D_{m}$ is diffusivity of microorganisms.

Using the below mentioned transformations

7 $$\begin{aligned} {\bar{u}}= & {} {\bar{U}}_{0}e^{\frac{x}{L}}f^{\prime }\left( \eta \right) ,\text { } {\bar{v}}=-\left( \frac{\upsilon {\bar{U}}_{0}}{2L}\right) ^{\frac{1}{2}}e^{ \frac{x}{2L}}\left( f+\eta f^{\prime }\right) ,\text { }\eta =\left( \frac{ {\bar{U}}_{0}}{2\upsilon L}\right) ^{\frac{1}{2}}e^{\frac{x}{2L}}y, \nonumber \\ \theta= & {} \frac{{\bar{T}}-{\bar{T}}_{\infty }}{{\bar{T}}_{w}-{\bar{T}}_{\infty }}, \text { }\phi =\frac{C-C_{\infty }}{C_{w}-C_{\infty }},\text { }\xi =\frac{ n_{1}-n_{1\infty }}{n_{1w}-n_{1\infty }}, \end{aligned}$$

the continuity equation is identically satisfied and Eqs. (2–6) becomes

8 $$\begin{aligned} (1-n)f^{\prime \prime \prime }-2(f^{\prime })^{2}+ff^{\prime \prime }+nWef^{\prime \prime }f^{\prime \prime \prime }-M^{2}f^{\prime }+\lambda [\theta -Nr\phi -Rb\xi ]=0, \end{aligned}$$

9 $$\begin{aligned} \theta ^{\prime \prime }+\Pr f\theta ^{\prime }+\Pr Nt\left( \theta ^{\prime }\right) ^{2}+\Pr Nb\phi ^{\prime }\theta ^{\prime }+\Pr M^{2}Ec(f^{\prime })^{2}=0, \end{aligned}$$

10 $$\begin{aligned} \phi ^{\prime \prime }+Lef\phi ^{\prime }+\frac{Nt}{Nb}\theta ^{\prime \prime }=0, \end{aligned}$$

11 $$\begin{aligned} \xi ^{\prime \prime }+Lbf\xi ^{\prime }-Pe(\xi +1)\phi ^{\prime \prime }-Pe\xi ^{\prime }\phi ^{\prime }]=0, \end{aligned}$$

12 $$\begin{aligned} \left. f(0)=0,\text { \ }f^{\prime }\left( 0\right) =1,\text { \ }\theta (0)=1,\text { \ }Nb\text { }\phi ^{\prime }(0)+Nt\text { }\theta ^{\prime }(0)=0,\text { \ }\xi (0)=1,\right. \nonumber \\ \left. \text { \ }f^{\prime }\left( \infty \right) \rightarrow 0,\text { \ \ \ }\theta (\infty )\rightarrow 0,\text { \ \ }\phi (\infty )\rightarrow 0, \text { \ \ }\xi (\infty )\rightarrow 0.\right. \end{aligned}$$

The dimensionless parameters are

13 $$\begin{aligned} \lambda= & {} \frac{2Lg\beta _{T}(1-{\bar{C}}_{\infty }){\bar{\rho }}_{f}\left( {\bar{T}}_{w}-{\bar{T}}_{\infty }\right) }{U_{0}^{2}},\text { \ }M^{2}=\frac{2L\sigma B_{0}^{2}e^{-\frac{x}{L}}}{{\bar{\rho }}_{f}U_{0}},\text { \ }We=\frac{\sqrt{2} \Gamma U_{0}^{\frac{3}{2}}e^{\frac{3x}{2L}}}{\sqrt{\upsilon L}}, \nonumber \\ Nr= & {} \frac{\left( {\bar{C}}_{w}-{\bar{C}}_{\infty }\right) ({\bar{\rho }}_{p}-{\bar{\rho }}_{f})}{\beta \rho _{f}\left( 1-{\bar{C}}_{\infty }\right) {\bar{T}}_{\infty }},\text { \ }R_{b}=\frac{({\bar{\rho }}_{m}-{\bar{\rho }}_{f})\gamma \left( {\bar{n}} _{1w}-{\bar{n}}_{1\infty }\right) }{{\bar{\rho }}_{f}\left( 1-{\bar{C}}_{\infty }\right) \beta \left( {\bar{T}}_{w}-{\bar{T}}_{\infty }\right) },\text { \ }Nt= \frac{({\bar{\rho }}c)_{p}{\bar{D}}_{T}\left( {\bar{T}}_{w}-{\bar{T}}_{\infty }\right) }{\upsilon ({\bar{\rho }}c)_{f}}, \nonumber \\ Nb= & {} \frac{({\bar{\rho }}c)_{p}{\bar{D}}_{B}\left( {\bar{C}}_{w}-{\bar{C}}_{\infty }\right) }{\upsilon ({\bar{\rho }}c)_{f}},\text { \ }Le=\frac{\upsilon }{{\bar{D}} _{B}},\text { }Lb=\frac{\upsilon }{{\bar{D}}_{m}},\text { \ }Ec=\frac{ U_{0}^{2}e^{\frac{2x}{L}}}{c_{p}k\left( {\bar{T}}_{w}-{\bar{T}}_{\infty }\right) },\text { } \nonumber \\ P_{e}= & {} \frac{{\bar{b}}W_{c}}{{\bar{D}}_{m}},\text { \ }\Pr =\frac{\mu c_{p}}{ \kappa }. \end{aligned}$$

in which $\lambda$ represents mixed convective parameter, *M* represents magnetic number, *We* represents Weissenberg number, *Nr* represents buoyancy force number, *Nb* represents Brownian motion parameter, $R_{b}$ represents bioconvection Rayleigh number, *Lb* represents bioconvection Lewis number, *Nt* represents thermophoresis parameter, *Le* represents Lewis parameter, *Ec* represents Eckert parameter, $P_{e}$ represents bioconvective Pecelt parameter and $\Pr$ represents Prandtl parameter.

Dimensional SFC, LNN and LDMM become

14 $$\begin{aligned} C_{fx}=\frac{2\tau _{xy}}{\rho U_{w}^{2}},\text { \ }N_{ux}=\frac{xq_{w}}{ K\left( T-T_{\infty }\right) },\text { \ }N_{nx}=\frac{xq_{n}}{D_{n}\left( n-n_{\infty }\right) }, \end{aligned}$$

15 $$\begin{aligned} \tau _{xy}=\mu \left( \left( 1-n\right) \frac{\partial {\bar{u}}}{\partial y} +2\Gamma n\left( \frac{\partial {\bar{u}}}{\partial y}\right) ^{2}\right) , \text { \ }q_{w}=\left. \frac{\partial T}{\partial y}\right| _{y=0},\text { \ }q_{n}=\left. \frac{\partial n_{1}}{\partial y}\right| _{y=0}. \end{aligned}$$

The dimensionless form of SFC, LNN and LDMM are

16 $$\begin{aligned}&\left( \frac{\text{ Re }}{2}\right) ^{1/2}C_{fx}=\left( 1-n\right) f^{\prime \prime }\left( 0\right) +\frac{n}{2}We\left( f^{\prime \prime }\left( 0\right) \right) ^{2}. \end{aligned}$$

17 $$\begin{aligned}&\left( \frac{\text{ Re}_{x}}{2}\right) ^{-1/2}N_{ux} =-\theta ^{\prime }(0), \nonumber \\&\left( \frac{\text{ Re}_{x}}{2}\right) ^{-1/2}N_{nx} =\xi ^{\prime }(0), \end{aligned}$$

where $\text{ Re}_{x}=\frac{U_{0}Le^{\frac{x}{L}}}{\upsilon }$ is the local Reynold number.

## Model selection: AIC and BIC

Model selection process are guidelines that are used to choose a statistical model from a list of candidates depending on data. The first broad metric for selecting models estimated by maximum likelihood was proposed by Akaike^35^. The AIC is the most commonly used model selection method in statistics. One can determine the best fit for the data by calculating and comparing the AIC scores of various different models. Using the maximum likelihood estimate and the number of parameters in the model, AIC calculates the relative information value of the model. This criterion, is widely regarded as the first model selection criterion to be employed in practise.The Bayesian Information Criterion, or BIC for short, is another prominent model selection criterion. Bayesian probability and inference is the subject of research from which it was derived. It’s appropriate for models that fit within the maximum likelihood estimation framework, just like AIC. Other prominent model selection methods include the AIC corrected for small-sample bias (AICc) and the Hannan-Quinn criterion (HQC). The data for the SFC, LNN and LDMM were tested using Akaike information criterion (AIC) and the Bayesian Information Criterion (BIC). These test are utilized to determine goodness of fit and find the model that fits best to data. The different statistical models are mentioned in Table 1. The AIC and BIC were determined for each model in the table, and the best distribution was identified from the values of AIC and BIC. The AIC/BIC determines the quality of statistical distributions for a sample set of data. The model which gives the lowest BIC/AIC value best fits the data. The formula for the AIC and BIC are

18 $$\begin{aligned} AIC=-2\log \left( L\right) +2k, \end{aligned}$$

where *k* is the number of estimated parameters and *L* is the maximized likelihood function in the model.

19 $$\begin{aligned} BIC=-2\log \left( L\right) +k\log \left( n\right) . \end{aligned}$$

## Discussion

The shooting methodology was utilized to achieve the numerical simulation for (8)–(11) with boundary conditions (12). The shooting techniques transform a BVP (boundary value problem) into an IVP (initial value problem). This methodology was employed by using “dsolve” command and the “shoot” implementation in Mathematica programming language. The influences of *Nr*, *Nt*, and *Lb* on the SFC, LNN and LDMM were investigated.

**Figure 2:**
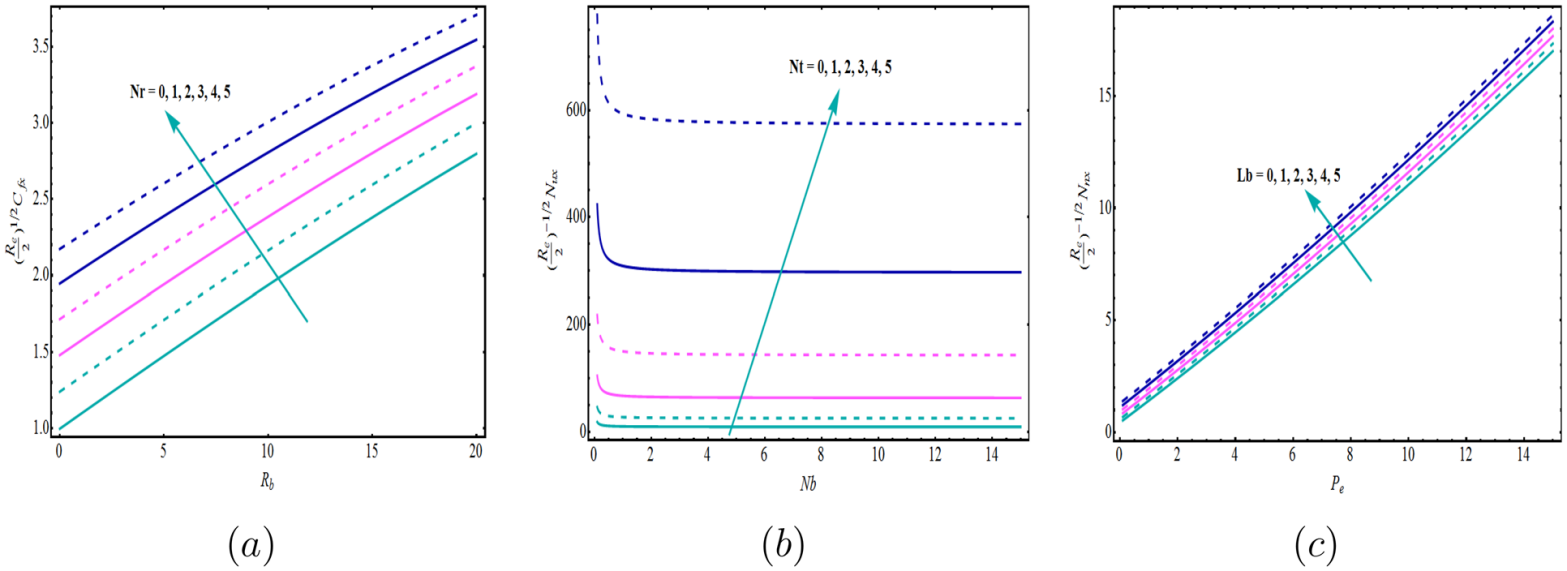
Variations for (**a**) SFC, (**b**) LNN, (**c**) LDMM with $R_{b}$, *Nb*,  $P_{e}$ with various values of *Nr*,  *Nt* and $P_{e}$, respectively.

**Figure 3:**
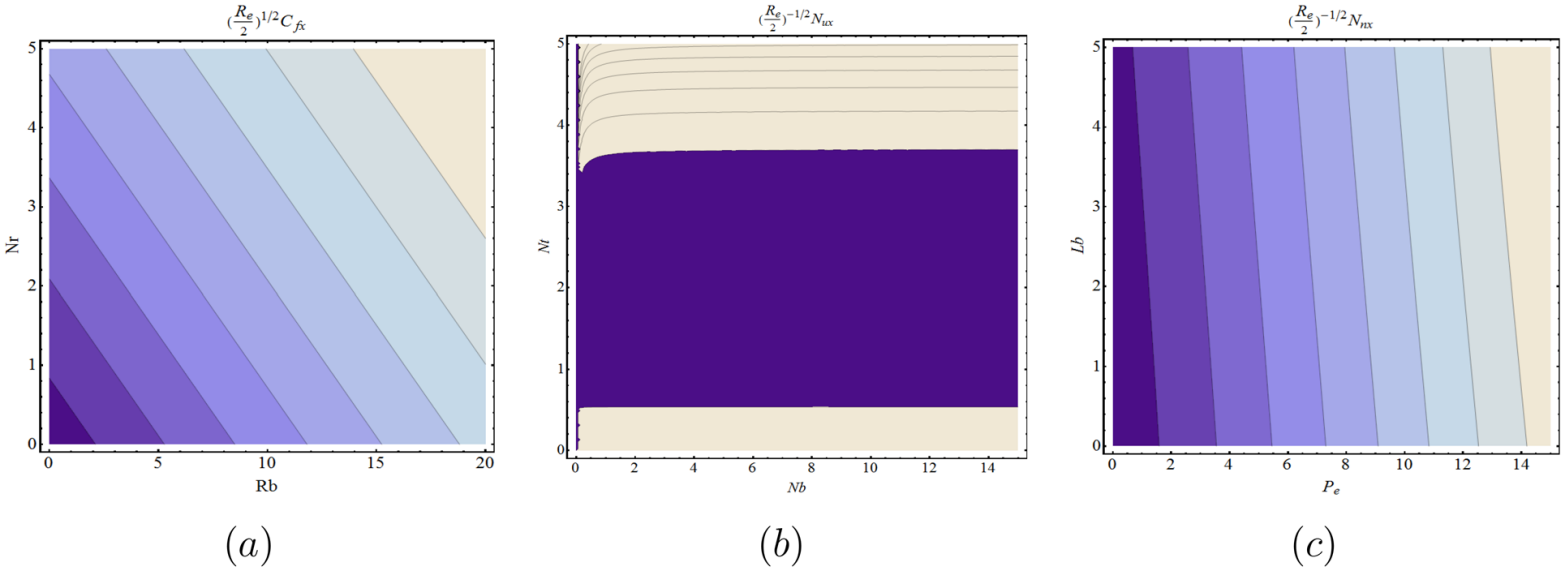
Contour graphs for (**a**) SFC, (**b**) LNN, (**c**) LDMM with $R_{b}$, *Nb*,  $P_{e}$ with various values of *Nr*,  *Nt* and $P_{e}$, respectively.

Figure 2a–c depicts the variation in the SFC, LNN and LDMM for different significant physical parameters. It is observed from Fig. 2a that as *Nr* increases, SFC increases, while Fig. 2b clearly shown that LNN is increasing function of *Nt* when *Nb* ranges from 0 to 15. Figure 2c is plotted for the various values of Lb for LDMM when $0\le P_{e}\le 15$. Figure 2c shows the same trend for *Lb*. It is worth remembering that the Brownian motion parameter *Nb* and the thermophoresis *Nt* are associated with the nanoparticles’ random motion. For smaller values of *Nb* and *Nt*, the fluid viscosity is low, and the nanomaterials and microorganisms tend to pass easily between each other. The fluid is cooled faster because of this phenomenon, and heat transfer rate increases. The contour plot is sketched for the same parameters corresponds to SFC, LNN and LDMM (see Fig. 3a–c).

The data for the skin friction coefficient, Nusselt and density of motile microorganisam numbers were further analysed on the basis of Fig. 2, in order to obtain the statistical properties for the tested models. Tables 2, 3 and 4 present the estimated parameters of the different distributions that have been tested with the considered data. Tables 5, 6 and 7 demonstrate the Akaike Information Criteria (AIC) and Bayesian Information Criterion (BIC) for the SFC, LNN and LDMM numbers.

**Figure 4:**
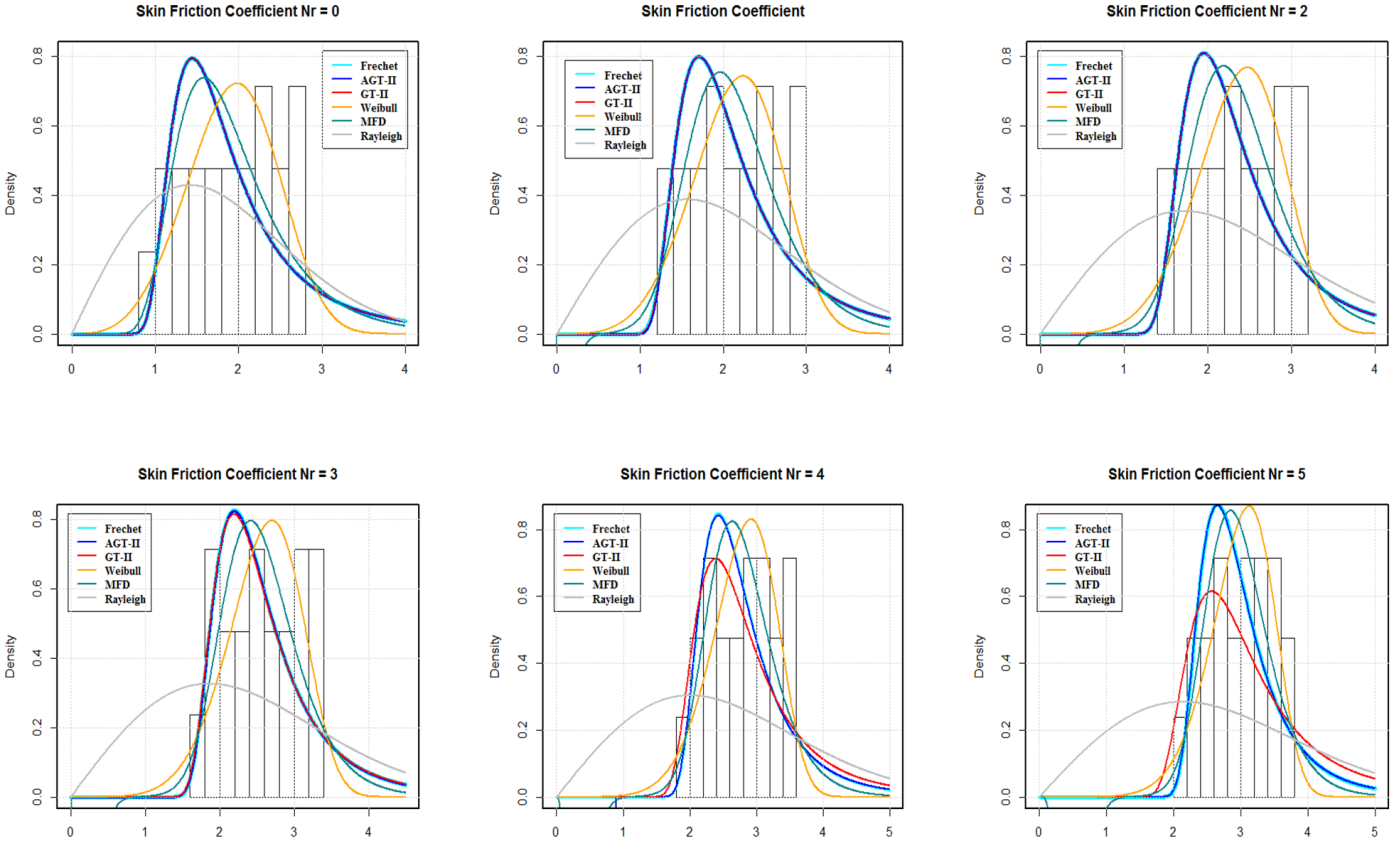
The estimated densities for the SFC.

**Figure 5:**
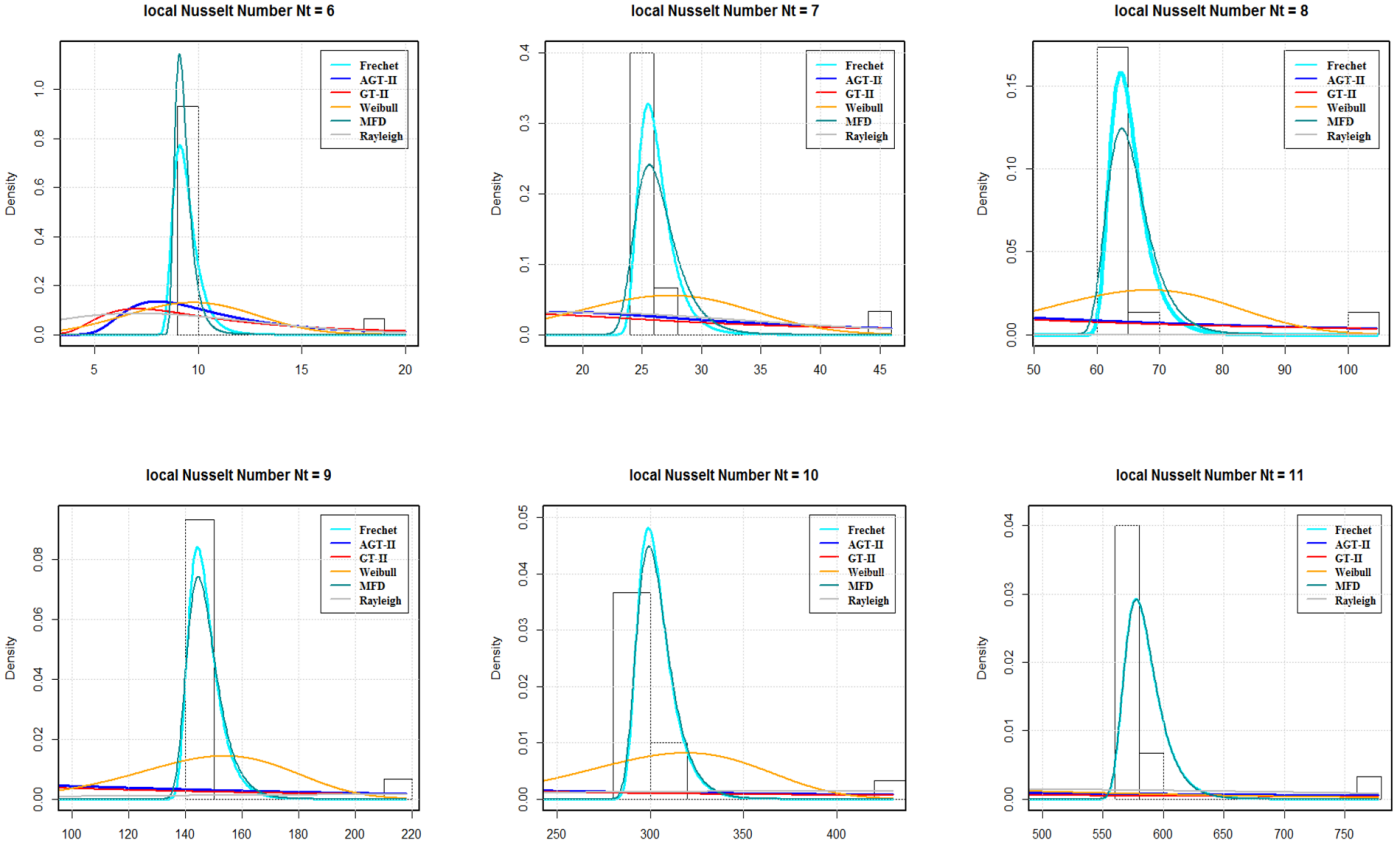
The estimated densities for the LNN.

**Figure 6:**
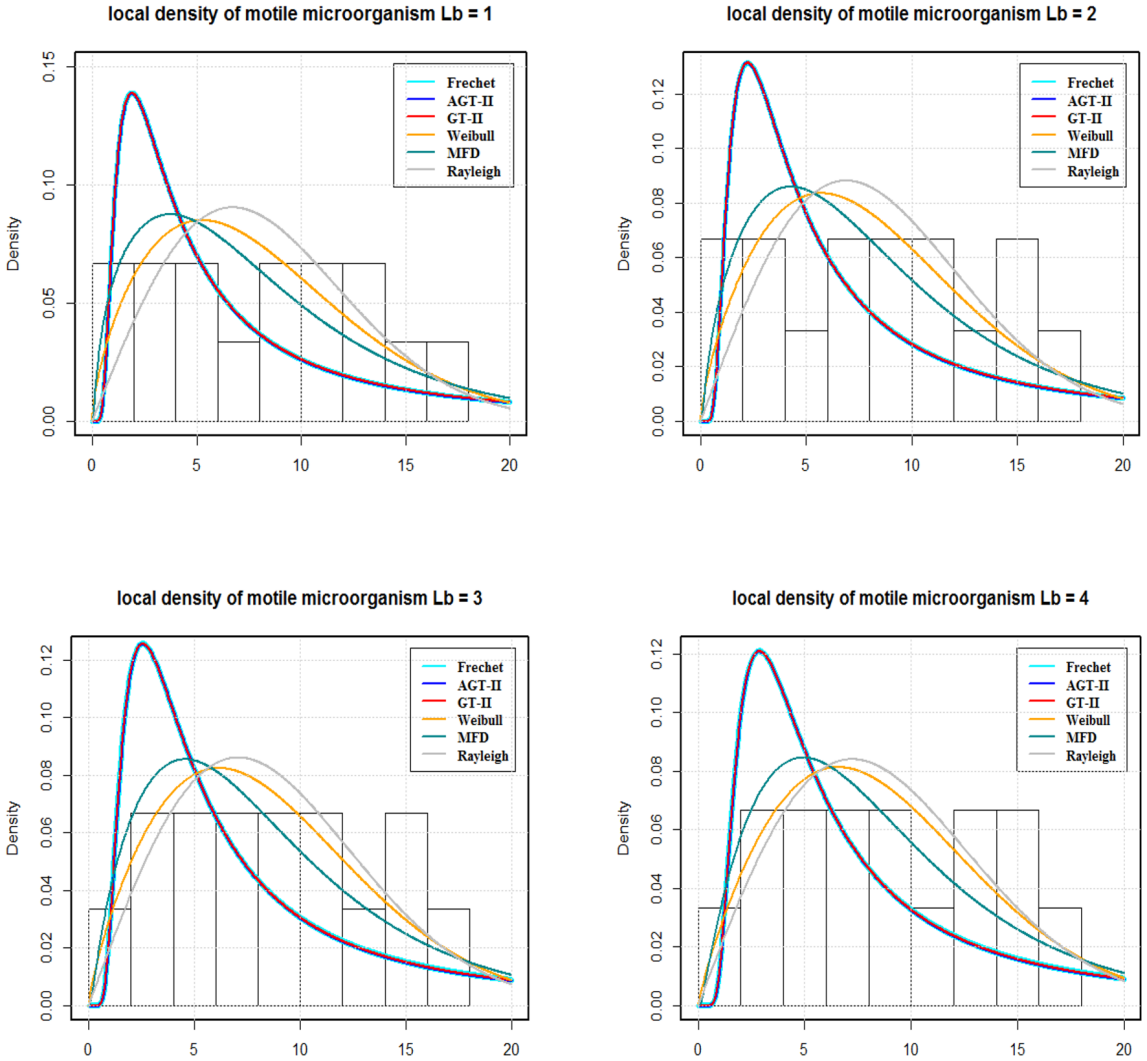
The estimated densities for the LDMM.

By using AIC and BIC as the model selection criteria, it is noticed that the Weibull distribution is suitable for modelling the SFC and LDMM (see Tables 5, 7). On the other side, for LNN, Frechet distribution is suitable under both AIC and BIC criteria. The estimated densities using data of SFC, LNN and LDMM under abovementioned models (see Figs. 4, 5, 6). Through these figures it can be observed that the Weibull distribution is best fitted model for SFC and LDMM. While, Frechet distribution is best fitted for LNN.

**Table 1:** The distribution test for SFC, LNN and LDMM.

Distribution	Probability distribution function $f\left( x\right)$
Frechet distribution	$$f\left( x;\alpha ,\beta \right) =\frac{\beta }{x}\left( \frac{\alpha }{x}\right) ^{\beta }e^{-\left( \frac{\alpha }{x}\right) ^{\beta }}$$$$x>0,$$ $$\alpha ,\beta >0,$$
	where $\alpha ,$ $\beta$ are shape parameters.
Additive Gumbel Type II Distribution	$f\left( x;\eta _{i},\delta _{i}\right) =\sum _{i=1}^{2}\eta _{i}\delta _{i}x^{-\eta _{i}-1}e^{-\sum _{i=1}^{2}\delta _{i}x^{-\eta _{i}}}$ $\ \ x,$ $\left( \delta _{i},\eta _{i}\right) >0$
	Where the shape parameters are $\delta _{i},$ $\eta _{i}$, $i=1,2.$
Gumbel Type II Distribution	$f\left( x;\eta ,\delta \right) =\eta \delta x^{-\eta -1}e^{-\delta x^{-\eta }}$ $\ \ \left( x,\delta ,\eta \right) >0,$
	Where the shape parameters are $\delta ,$ $\eta$.
Weibull Distribution	$$f\left( x;\lambda ,\beta \right) =\frac{\beta }{\lambda }\left( \frac{x}{\lambda } \right) ^{\beta -1}e^{-\left( \frac{x}{\lambda }\right) ^{\beta }}$$$$x>0,$$ $$\lambda ,\beta >0,$$
	where $\beta >0$ is the shape parameter and $\lambda >0$
	is the scale parameter of the distribution.
Modified Frechet distribution	$f\left( x;\alpha ,\beta ,\lambda \right) =\frac{1}{x}\left( \beta +\lambda x\right) \left( \frac{\alpha }{x}\right) ^{\beta }\exp \left[ -\lambda x-\left( \frac{\alpha }{x}\right) ^{\beta }\right]$
	$x>0,$ $\alpha ,\beta >0,$ $\ \lambda \ge 0,$
	where $\alpha ,$ $\beta ,$ $\lambda$ are shape parameters.
Rayleigh Distribution	$f\left( x;\sigma \right) =\frac{x}{\sigma ^{2}}\exp \left[ -\frac{x^{2}}{2\sigma ^{2} }\right]$
	where $\sigma$ is the scale parameter of the distribution.

**Table 2:** Estimates of the parameters of statistical distribution for SFC.

		SFC					
		*Nr*					
		0	1	2	3	4	5
FD	${\hat{\alpha }}$	1.568950	1.812761	2.048765	2.277253	2.498126	2.711093
	${\hat{\beta }}$	3.249076	3.819011	4.407746	5.023453	5.674475	6.370279
	${\hat{\eta }}_{1}$	2.340444	9.769603	14.49602	61.68615	-3.898806	8.543967
AGT-II	${\hat{\delta }}_{1}$	1.980203	2.195986	9.106387	38.38976	173.47197	565.10015
	${\hat{\eta }}_{2}$	3.249075	3.820303	4.407449	5.008879	37.15317	23.634421
	${\hat{\delta }}_{2}$	3.249069	14.637496	4.408063	32.58794	5.630879	6.353374
GT-II	${\hat{\eta }}$	3.249075	3.818910	4.404736	4.949803	4.720524	4.381971
	${\hat{\delta }}$	4.320648	9.696103	23.54969	58.52508	73.90224	75.942714
Weibull	${\hat{\lambda }}$	4.038674	4.644257	5.278253	5.950158	6.670540	7.452207
	${\hat{\beta }}$	2.126016	2.354407	2.574283	2.785597	2.988088	3.181357
	${\hat{\alpha }}$	27.63889	0.0636608	0.1125029	0.182326	0.2705209	0.3647409
MFD	${\hat{\beta }}$	0.847507	-1.6221723	-2.2936863	-3.249356	-4.5623501	-6.2105315
	${\hat{\lambda }}$	1.480263	2.8919409	3.1625153	3.528906	3.9943797	4.5362588
RD	${\hat{\sigma }}$	1.412951	1.564143	1.712606	1.857528	1.998237	2.134108

**Table 3:** Estimates of the parameters of statistical distribution for LNN.

		LNN					
		*Nt*					
		6	7	8	9	10	11
FD	${\hat{\alpha }}$	9.140826	25.54203	63.89860	144.3362	299.1832	577.6288
	${\hat{\beta }}$	19.18480	22.74168	27.43727	32.98885	39.12873	45.97684
	${\hat{\eta }}_{1}$	78.15356	99.97835	144.78854	213.8291	135.2225	9.497816
AGT-II	${\hat{\delta }}_{1}$	97.92131	84.87295	137.58036	206.7033	125.0395	2.050675
	${\hat{\eta }}_{2}$	2.394169	1.733487	1.373428	1.243163	0.987689	3.005450
	${\hat{\delta }}_{2}$	2.398008	1.618335	1.434913	1.247597	0.993976	1.217642
GT-II	${\hat{\eta }}$	2.171243	1.453281	1.202308	0.972356	0.827369	0.740383
	${\hat{\delta }}$	101.4447	94.73049	125.691247	106.4941	87.12970	85.61986
Weibull	${\hat{\lambda }}$	3.618602	4.251510	5.089174	6.074484	7.143974	1.756068
	${\hat{\beta }}$	10.66843	29.12578	71.293623	158.2191	325.7668	369.0693
	${\hat{\alpha }}$	0.025798	0.036827	0.05373844	1.331674	1.0511818	4.845107
MFD	${\hat{\beta }}$	-2.746046	-3.022394	-3.55584431	-7.889381	-7.8376355	-12.11829
	${\hat{\lambda }}$	1.7597250	0.773008	0.39368449	0.256016	0.1480056	0.100307
RD	${\hat{\sigma }}$	420.0692	3313.579	1140.704	338.1586	432.1877	419.0887

**Table 4:** Estimates of the parameters of statistical distribution for LDMM.

		LDMM			
		*Lb*			
		1	2	3	4
F-D	${\hat{\alpha }}$	3.875291	4.192726	4.498246	4.795930
	${\hat{\beta }}$	0.981468	1.053006	1.122236	1.188403
	${\hat{\eta }}_{1}$	1.890055	2.260506	2.702876	3.220559
AGT-II	${\hat{\delta }}_{1}$	1.890055	2.260506	2.702876	3.220559
	${\hat{\eta }}_{2}$	0.981294	1.052955	1.122235	1.188242
	${\hat{\delta }}_{2}$	0.981294	1.052955	1.122225	1.188242
GT-II	${\hat{\eta }}$	0.981294	1.052957	1.122196	1.188337
	${\hat{\delta }}$	3.780108	4.521043	5.406437	6.443316
Weibull	${\hat{\lambda }}$	1.663800	1.725847	1.786556	1.845495
	${\hat{\beta }}$	9.108319	9.423109	9.734224	10.03794
	${\hat{\alpha }}$	58.31327	79.70721	73.15271	89.53789
MFD	${\hat{\beta }}$	0.336251	0.332175	0.358084	0.359264
	${\hat{\lambda }}$	0.161677	0.165079	0.162227	0.163958
RD	${\hat{\sigma }}$	6.710372	6.880247	7.050128	7.217048

**Table 5:** Akaike information criteria (AIC) and (BIC) for SFC.

		SFC					
		*Nr*					
		0	1	2	3	4	5
F-D	AIC	43.71452	42.19349	40.68414	39.11847	37.4521	35.64968
	BIC	45.80357	44.28254	42.77318	41.20752	39.54114	37.73873
AGT-II	AIC	47.71452	46.1758	44.68414	43.11883	41.45449	39.64996
	BIC	51.89261	50.35389	48.86223	47.29692	45.63258	43.82805
GT-II	AIC	43.71452	42.19349	40.68416	39.12911	38.76079	40.4071
	BIC	45.80357	44.28254	42.7732	41.21815	40.84983	42.49614
Weibull	AIC	**37.59644**	**36.69447**	**35.62046**	**34.36348**	**32.91114**	**31.24644**
	BIC	**39.68548**	**38.78351**	**37.70951**	**36.45252**	**35.00019**	**33.33548**
MFD	AIC	42.44299	40.75795	39.58348	38.25849	36.76681	35.09028
	BIC	45.57655	43.89152	42.71705	41.39206	39.90037	38.22384
RD	AIC	47.46089	50.88832	54.10217	57.07796	59.81554	62.32395
	BIC	48.50541	51.93284	55.14669	58.12248	60.86006	63.36847

**Table 6:** Akaike information criteria (AIC) and (BIC) for the LNN.

		LNN					
		*Nt*					
		6	7	8	9	10	11
F-D	AIC	**39.59408**	**65.19384**	**86.95979**	**105.8262**	**122.4597**	**137.3348**
	BIC	**41.01018**	**66.60994**	**88.37589**	**107.2423**	**123.8758**	**138.7509**
AGT-II	AIC	81.71779	122.5622	154.628	182.0513	210.2601	223.8249
	BIC	84.54999	125.3944	157.4602	184.8835	213.0923	226.6571
GT-II	AIC	80.71941	122.2521	154.9725	185.4515	212.4454	235.4214
	BIC	82.13551	123.6682	156.3886	186.8676	213.8615	236.8375
Weibull	AIC	74.96524	100.7408	122.6578	141.6394	158.4089	222.6907
	BIC	76.38134	102.1569	124.0739	143.0555	159.8250	224.1068
MFD	AIC	51.74538	75.66230	95.93718	114.3271	129.8048	144.3277
	BIC	53.86953	77.78645	98.06133	116.4513	131.9289	146.4519
RD	AIC	296.6721	389.9711	298.727	204.4000	202.0818	202.9555
	BIC	297.3802	390.6792	299.435	205.1081	202.7899	203.6636

**Table 7:** Akaike information criteria (AIC) and (BIC) for LDMM.

		LDMM			
		*Lb*			
		1	2	3	4
F-D	AIC	103.3815	102.9313	102.5711	102.2829
	BIC	104.7976	104.3474	103.9872	103.6990
AGT-II	AIC	107.3815	106.9313	106.5711	106.2829
	BIC	110.2137	109.7635	109.4033	109.1151
GT-II	AIC	103.3815	102.9313	102.5711	102.2829
	BIC	104.7976	104.3474	103.9872	103.699
Weibull	AIC	**92.57561**	**92.8465**	**93.0999**	**93.33274**
	BIC	**93.99171**	**94.2626**	**94.516**	**94.74884**
MFD	AIC	95.8456	96.16195	96.54202	96.80884
	BIC	97.96975	98.2861	98.66617	98.93299
RD	AIC	91.35215	91.34132	91.3873	91.47751
	BIC	92.0602	92.04937	92.09535	92.18556

## Concluding remarks

This present study investigate implementation of a numerical solution of bioconvective nanofluid flow. The boundary layer flow (BLF) towards a vertical exponentially stretching plate with combination of heat and mass transfer rate in tangent hyperbolic nanofluid containing microorganisms. We have introduced zero mass flux condition to achieve physically realistic outcomes. Analysis is conducted with magnetic field phenomenon. By using similarity variables, the partial differential equation which governs the said model was converted into a nonlinear ordinary differential equation, and numerical results are achieved by applying the shooting technique. Bioconvective nanoliquid stream towards an expending surface and impacts of parameters *Nr*,  *Rb*,  *Lb*,  *Pe*,  *Nt* and *Nb* is analyzed and studied. From this study, we obtain a unique solution for expanding surface. It is noted that, as *Nr* and *Rb* increases, the skin friction coefficient rises. The rate of mass transfer is increased by increasing *Lb* and *Pe*. Furthermore, for least values of *Nb* and all the values of Nt under consideration the heat transfer rate upsurges. The data of SFC, LNN, and LDMM have been tested utilizing various statistical models, and it is noted that data sets for SFC and LDMM fit the Weibull model for different values of *Nr* and *Lb* respectively. On the other hand, Frechet distribution fits well for LNN data set for various values of *Nt*.
